# Physico-functional and nutritional characteristics of germinated pigeon pea (*Cajanus cajan*) flour as a functional food ingredient

**DOI:** 10.1038/s41598-023-43607-8

**Published:** 2023-10-03

**Authors:** Richard Atinpoore Atuna, Mary-Ann Sarpong Mensah, Gifty Koomson, Fortune Akabanda, Selorm Yaotse Dorvlo, Francis Kweku Amagloh

**Affiliations:** 1 Department of Food Science and Technology, Faculty of Agriculture, Food and Consumer Sciences, University for Development Studies, Tamale, Ghana; 2 Agricultural Engineering Department, University of Ghana, Legon, Ghana

**Keywords:** Biotechnology, Biomaterials

## Abstract

The study investigated the effect of germination on pigeon pea flour’s physico-functional (pH, color*,* water and oil absorption capacities, swelling and foaming capacities and bulk densities) and proximate, total polyphenols and antioxidant activity. The physico-functional and proximate parameters were determined using standard protocols. The color analysis showed that germination significantly increased the flour samples’ lightness (L*) (70.7; p = 0.009) by almost 1.5-fold. Germination resulted in almost 1.1 times higher oil absorption capacity than the control (219.9%; p = 0.022). The foaming capacity of the germinated samples significantly (p = 0.015) increased by 6.4%. Germination significantly reduced the loose bulk density (0.54 *vs* 0.63; p = 0.012) but significantly increased the tapped bulk density (0.84 *vs* 0.77; p = 0.002). The germinated samples recorded significantly (1.62%; p = 0.010) lower crude fat, about 1.2 times lower than the raw flour. Germination significantly increased the flour’s total ash (4.2% *vs* 3.6%; p = 0.003) and crude protein (11.6% *vs* 9.4%; p = 0.047) content. Germinated pigeon pea flour will perform better in formulating baked products, aerated foods and food extenders than non-germinated pigeon pea flour. Hence, the germination of pigeon peas should be encouraged because it harnesses the functional and proximate attributes measured.

## Introduction

Childhood malnutrition (undernutrition and overnutrition) is a global problem^1^, with significant health and socio-economic implications^2^, and is the leading cause of illness and death worldwide^3^. Malnutrition or hidden hunger negatively impacts physical and mental growth, the immune system, and general health, limiting one’s ability to achieve their full potential^4^. Malnutrition affects one out of every three people, and almost every nation in Sub-Saharan Africa (SSA) is dealing with a significant public health problem due to malnutrition^5^.

Over the years, Ghana has made marginal gains in reducing malnutrition among pre-schoolers. Nevertheless, malnutrition continues to be a significant  public health concern in rural parts of Ghana. To illustrate, in the Northern region, 29% of children younger than 5 years are moderately or severely stunted (too short for their age.), and 11% are wasting (too thin for their height)^6^. The attributable cause of this poor statistic in rural Ghana is the inability of poor households to afford diversified diets to meet their nutritional needs due to poverty. In addition, the over-dependence on cheap energy-dense, nutrient-poor staples with high levels of anti-nutritional factors also contribute to the burden of hidden hunger.

The utilization of legumes in food-to-food fortification has been recognized as a sustainable approach to enhance the traditionally nutrient-deficient cereal-based diets, effectively tackling the problem of malnutrition in developing nations^7^. Legumes, particularly pigeon pea, is rich in protein, minerals, and phytochemicals, making them an essential and inexpensive food staple in the Global South^8^.

Despite the nutritional potentials of pigeon pea in addressing hidden hunger, the key limitation for their utilization and consumption in our daily diet include poor digestibility and the presence of anti-nutrients, such as phytic acids, trypsin inhibitors, oligosaccharides and phenolic compounds^9^.

Nevertheless, adequate processing methods can be used to minimize the anti-nutrients and improve the digestibility and bioavailability^10^ without much effort. Traditional household food processing techniques such as fermentation, germination/malting, soaking and thermal processes have been previously employed to improve the nutritional quality of cereals, legumes/pulses, and root tubers or minimize the levels of anti-nutrients^11^. The application of such technological processes provokes changes in the physicochemical characteristics of the components^9^. In particular, germination triggers endogenous enzymes to modify the grain’s constituents^12,13^. Germination has been reported to be most promising in this regard, resulting in desirable changes in consistency and nutritional properties^11^. It is simple, less expensive, culturally acceptable and can be employed at the household level without much demand on time, labor or space^14,15^. Germination offers numerous nutritional and organoleptic advantages^16,17^ because it helps synthesize vitamin C and converts starch to simple sugar^18^. There has been some evidence of the enhancement in the availability of proteins^19,20^, carbohydrates^20^, vitamins^21^ and minerals^10,22^ with germination. The reduction of several anti-nutritional factors using germination has also been documented^23–26^, while some evidence also suggests a reduction in viscosity by germination^27^, a desirable attribute for complementary food ingredients.

Therefore, this study investigated the effect of germination on the physico-fucntional properties of pigeon pea flour as a functional food ingredient. Also, the nutritional qualities of the germinated pigeon pea flour and total polyphenols and antioxidant activity were considered. The study hypothesized that germination would improve the functional and nutritional properties and decrease the anti-nutrient content of pigeon peas. This will significantly improve their digestibility and nutrient bioavailability and consequently address the issue of malnutrition among pre-schoolers in Ghana and other developing countries. Besides, it will broaden the frontiers of pigeon pea utilization in Ghana.

## Results

**Figure 1:**
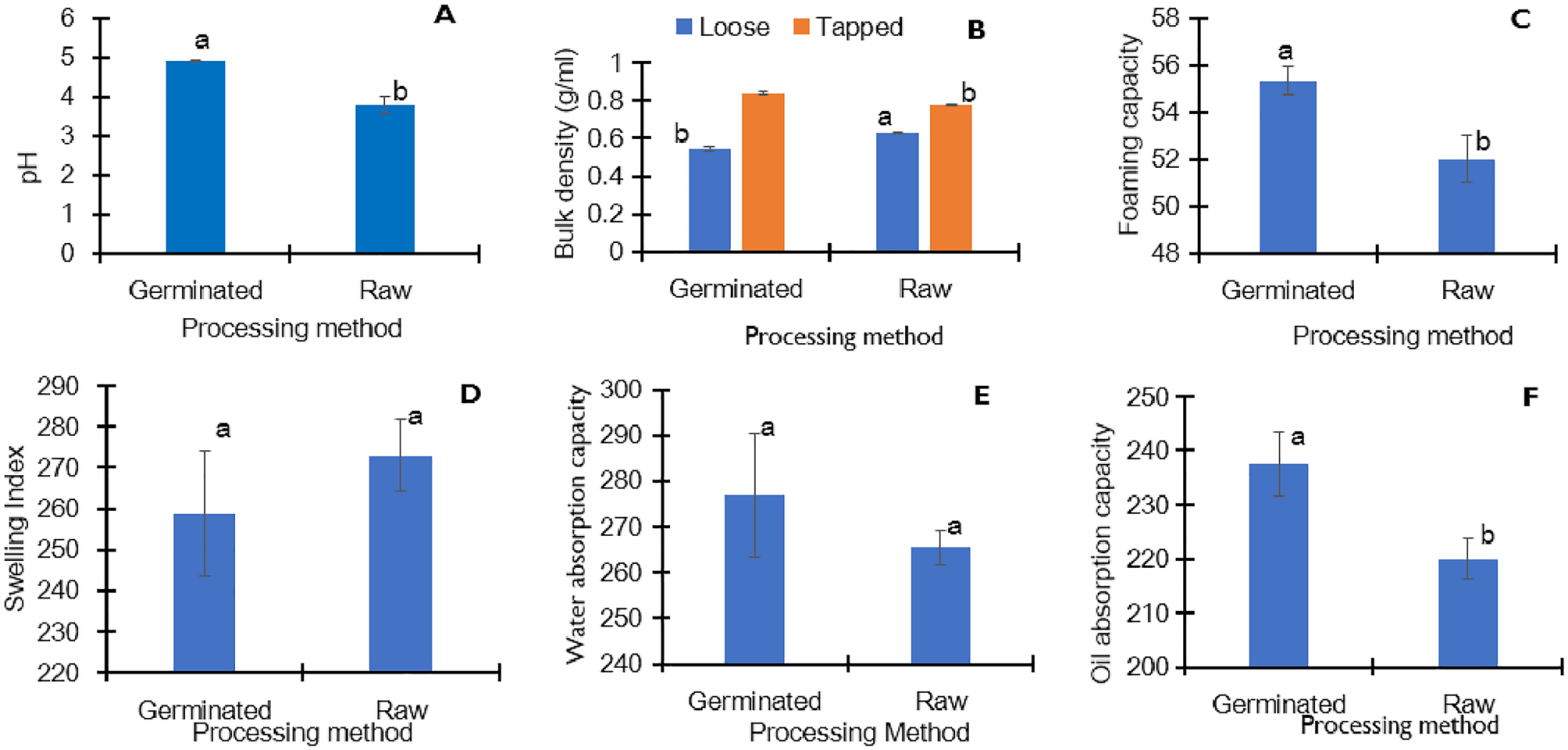
Physico-functional characteristics of germinated pigeon pea flour: pH (**A**), bulk density (**B**), foaming capacity (**C**), swelling index (**D**), water absorption capacity (**E**) and oil absorption capacity (**F**). Bar values are means ± SD; *n* = 3. Means with the same letters are not significantly (p > 0.05) different.

The physico-functional properties of germinated pigeon pea flour are presented in Fig. 1A–F. Germination resulted in a significant (4.9 vs 3.8; p = 0.013) increase in pH (Fig. 1A). The loose bulk density was significantly (p = 0.012) lower in the germinated pigeon pea flour compared to the raw flour (Fig. 1B). The germinated pigeon pea flour had a significantly (p = 0.015) higher foaming capacity, about a 6.4% increase compared to the raw samples (Fig. 1C). The foaming capacity corresponds to the ability of the protein to provide an interfacial area for foam formation. Thus, the quality of protein determines the extent of foaming capacity. The swelling index (Fig. 1D) and water absorption capacity (Fig. 1E) did not show any significant changes with germination. However, it significantly (p = 0.022) increased the oil absorption capacity by nearly 1.1-fold (Fig. 1F).

Germination significantly influenced all the color parameters evaluated. The germinated flour showed significantly (p = 0.009) higher luminosity compared to the raw samples. In contrast, the raw samples had significantly higher a* values denoting redness (Table 1). The b* that denotes the yellowness or greenness was higher in the germinated samples.

**Table 1:** Instrumental color analysis of germinated pigeon pea flour as influenced by germination.

Processing method	Color parameter			
	L*	a*	b*	C
Germinated	70.77 ± 3.48	2.69 ± 0.43	21.82 ± 0.64	21.99 ± 0.63
Raw	48.78 ± 0.74	5.68 ± 0.64	17.26 ± 0.30	18.18 ± 0.28
p-value	0.009	0.007	0.008	0.011

Values are means ± SD; *n* = 3. Color parameters: L*, lightness (0 = black, 100 = white); a*, redness/greenness (− a = green, + a = red); b*, blueness/yellowness (− b = blue, + b = yellow).

**Figure 2:**
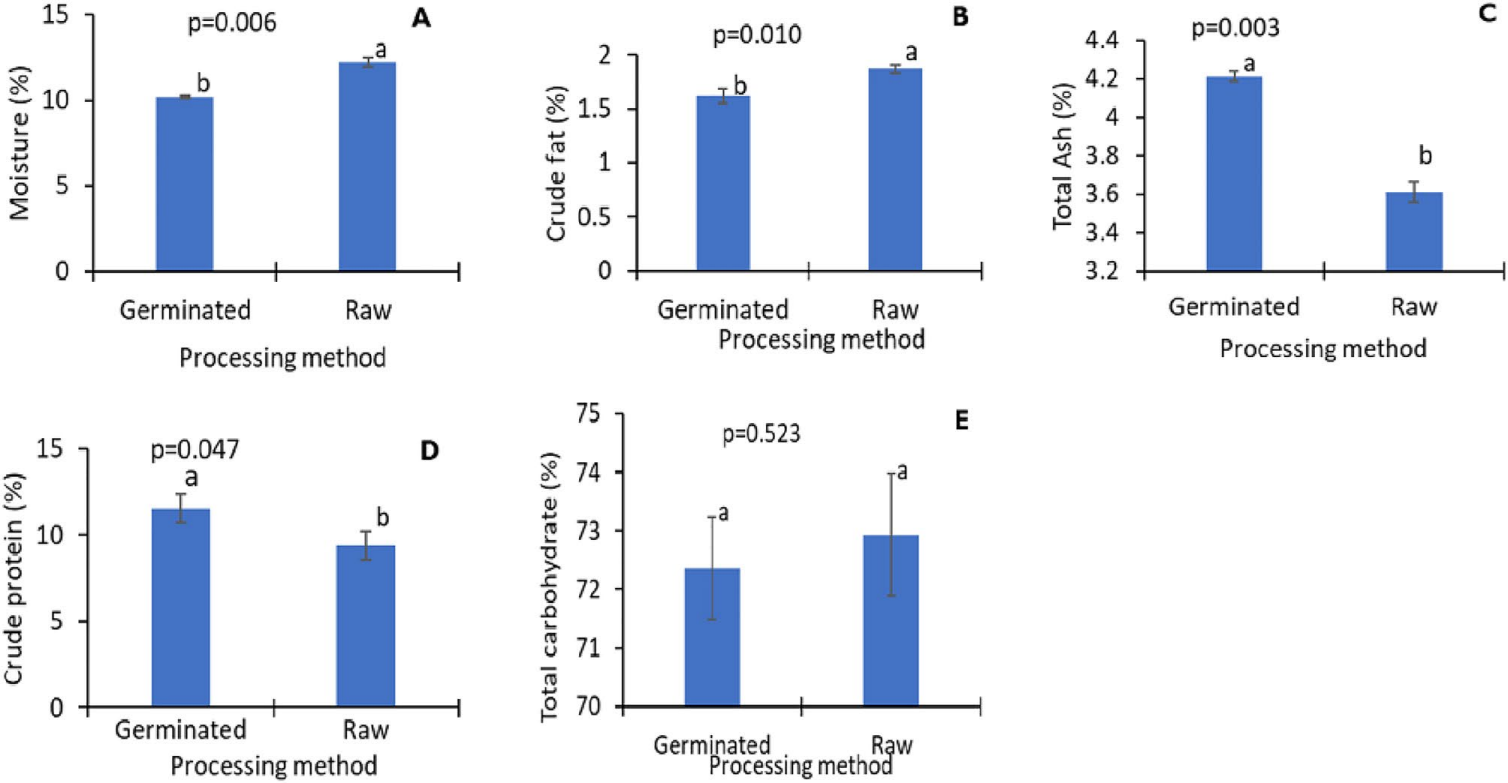
Proximate composition of germinated pigeon pea flour: Moisture (**A**); crude fat (**B**); total ash (**C**); crude protein (**D**) and total carbohydrates (**E**). Bar values are means ± SD; *n* = 3. Means with the same letters are not significantly (p > 0.05).

The germinated flour recorded significantly (p = 0.006) lower moisture content relative to the raw flour samples (Fig. 2A). The crude fat content of the germinated flour declined significantly (1.6% vs 1.9%; p = 0.010) by almost 1.2-fold compared to the raw samples (Fig. 2B). The total ash content markedly increased (p = 0.003) with germination (Fig. 2C). The increase in total ash content was about 1.2 times higher in the germinated flour samples than the raw samples. Also, the data in Fig. 2D showed that the crude protein content significantly increased (p = 0.047) by nearly 23.5% with germination. Although germination decreased total carbohydrate content, the decline was insignificant (p = 0.523), as presented in Fig. 2E.

**Figure 3:**
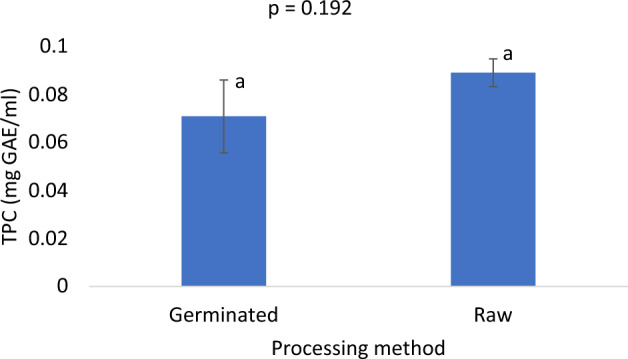
Total polyphenols content of germinated pigeon pea flour *vs* raw flour. Bar values are means ± SD; n = 3. Means with the same letters are not significantly (p > 0.05) different.

**Figure 4:**
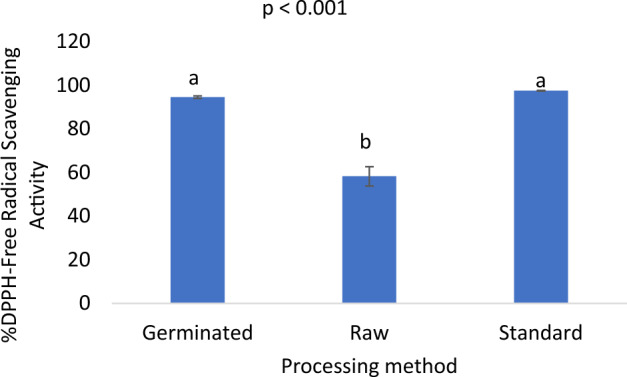
Free-radical scavenging activity of germinated pigeon pea flour. Bar values are means ± SD; *n* = 3. Means with the same letters are not significantly (p > 0.05) different.

Although not significant (p = 0.192), germination resulted in reduced total polyphenols content (Fig. 3). However, the germinated samples, though with lower polyphenols content, had a similar antioxidant activity as the standard (ascorbic acid) but significantly (p < 0.001) higher antioxidant activity than the raw flour samples. The antioxidant activity in the germinated samples was about 1.6 times higher than in the raw samples (Fig. 4).

## Discussion

Although the pH of the germinated pigeon pea samples was still acidic, the gradual increase in pH of the samples could be due to the initiation of lactic acid fermentation moving towards alkaline fermentation because of the protein-rich nature of the substrate. Generally, microbial growth and diversity are affected by a myriad of factors including pH, and temperature among others. Alkaline fermentation involves the fermentation of protein-rich raw materials such as legumes and tends to increase the pH of the substrates^28,29^.

The loose bulk density, an important functional property of flours, measures the highest attainable compaction without compression. The decrease in bulk density with germination could be attributed to the lowering of heaviness and dispersibility of the flour particles due to the breakdown of complex compounds like starch and protein. Similar findings have been reported in germinated pigeon pea flour^30^ and chickpea protein isolate powder^31^. A lower bulk density is significant for flours meant for infant foods because samples could be prepared with a small amount of water yet provide the desired energy and nutrient densities and semi-solid consistency. Besides, the packaging requirements of the flour are determined by the bulk density, which is a function of the flour's particle size and moisture content.

The foaming capacity of flour is a measure of the amount of interfacial area created by whipping the flour^32^. The enhancement in the foaming capacity of the germinated pigeon pea flour samples, as observed in this study, corresponds to the ability of the protein to provide an interfacial area for foam formation and to stabilize it against gravitational and mechanical stresses^30^. The findings agree with previous works that reported increased foaming capacity with germination^30,31,33^. The improved foaming capacity in flours could be attributed to the hydrolysis of large peptides into smaller peptides and amino acids by proteolytic enzymes released during germination. Good foam capacity is a desired property for flours intended for use in the production of various baked products such as cakes and cookies. Also, it performs as a functional agent in other food formulations^34^.

The swelling capacity is the measure of the ability of starch to imbibe water and swell and also indicates the extent of associative forces in the starch granules^32^. Swelling capacity (index) is regarded as a quality measure in some food products such as bakery products. The high swelling capacity of flour is suitable for making dough with high elasticity as reported earlier^35^. Therefore, pigeon pea flours with high swelling capacity could be utilised effectively as functional ingredients for pastry products such as bread, pasta, and other viscous foods.

Water absorption capacity (WAC) is the amount of water taken up by flour to achieve the desirable consistency and create quality food product^32^. The high WAC indicates the usefulness of flour especially in food formulations involving dough due to its ability to absorb water and swell for improved consistency in food. Thus, the higher WAC of the flour samples will enable bakers to add more water to doughs, improving handling and maintaining freshness in product development. One advantage of high-water absorption is its influence on easy softening, and easy, as well as increased digestibility. However, the disadvantage is that it also increases water activity, which is likely to cause food spoilage.

The trend observed in the oil absorption capacity in this study could be ascribed to the protein quality, its surface hydrophobicity, and its ability to hold fat globules^36^. The oil absorption capacity of flour has been reported to be influenced mainly by the amount of hydrophilic, and lipophilic amino acids and their spatial arrangements^30^. Thus, the oil absorption capacity is a function of fat, binding the non-polar side chains of protein, and has been affected by the number of hydrophobic sites and protein–lipid–carbohydrate interactions. Increment in the oil absorption capacity after germination has previously been reported in germinated cowpea^33^, germinated wheat, brown rice and triticale^30^. Oil absorption capacity is an essential functional property that enhances the flavor and mouthfeel of food since oil is reported to act as a flavor retainer^37^. Therefore, this property is required in most food applications, such as bakery products, which require flavor retention and improvement of palatability^38^. In contrast, this flour with high oil absorption capacities, such as germinated pigeon pea as found in this study, may not be ideal for foods that are meant to be fried as the high oil absorption may have some dire economic and storage stability consequences.

Color is one of the most critical quality attributes for determining a food product’s overall quality since it considerably influences the consumer acceptability for their utilization of various products. As found in this study, the increased lightness and decrease in redness with germination disagree with earlier works that reported lightness and increased redness with pigeon pea germination^39^. The variety of pigeon pea used could account for the discrepancies in the findings. However, the increase in yellowness and chroma with germination could be due to the concentration of carotenoids.

The proximate composition (moisture, crude fat, total ash, crude protein and total carbohydrate) is an essential criterion for determining the nutritional values and quality of food^40^. In this regard, the proximate composition of germinated pigeon pea flour was compared with ungerminated (raw) pigeon pea flour. The relatively low moisture content of the germinated pigeon pea flour indicates superior keeping quality, as high moisture content predisposes samples to spoilage. This is because increased moisture content can enhance microorganisms’ activity, leading to flour deterioration.

The decline in crude fat content of the germinated flour agrees with earlier reports of about a 32% reduction in crude fat content of germinated pigeon pea flour^41^. The germination process evokes lipolytic enzymes that hydrolyze fat into fatty acid and glycerol and its subsequent oxidation for energy production, hence the reduction in fat into fatty acid and glycerol and its subsequent oxidation for energy production^41^, reducing the crude fat content in the germinated samples. Besides, the reduction in crude fat content could also be ascribed to the reduction of stored fat due to the high catabolic activities in seeds during germination.

The increase in total ash content of the germinated pigeon pea flour samples in this study corroborates earlier findings that showed an almost 6% increase in total ash content of pigeon pea due to germination^41^.

The increase in crude protein content of the germinated pigeon pea flour samples in this study supports other previous studies. For example, an increased crude protein content due to germination was also reported in pigeon pea^41^ chickpea^42^, mungbean^43^ and faba beans^44^. The rise in crude proteins may be attributed to the synthesis of proteases required for certain amino acids during protein synthesis^39^.

The decline in total carbohydrates with fermentation is consistent with earlier studies that reported decreased carbohydrate content in mung bean, chickpea and cowpea^45^. Another study also reported a 2.3% decrease in carbohydrate content after 24 h of germination in cowpea^46^. The decline in total carbohydrates could be due to the breakdown of complex carbohydrates by alpha-amylase into absorbable sugars that are further used by the growing seedling during the early stages of germination.

The slight decline in total polyphenols content in the germinated pigeon pea flour samples in this study could be due to the soaking of the grains in water before the germination process as soaking before germination was found to reduce the total polyphenols content in pigeon pea by nearly 4.6%^39^. Several studies have reported increased phenolic compounds with germination in grains^41,47^. However, in the current study, the solubilization of water-soluble polyphenols that may have leached into the soak water could plausibly explain the decline in total polyphenols content with germination.

Many previous works have reported a linear relationship between total polyphenols content and antioxidant activity. Thus, samples with higher total polyphenol content possess higher antioxidant activity. However, despite the relatively low total polyphenols content in the germinated pigeon pea flour samples, they showed superior antioxidant activity, using ascorbic acid as an index. It is reported that during germination, the amount of phenolic compounds increases due to the presence of more hydroxyl groups that can provide the required component as free radical scavengers, which enhances the antioxidant activity of germination legumes^48^. Samples with high free radical scavenging activity refers to flour that contains compounds known as antioxidants, which can help counteract oxidative stress in the body^49^. Oxidative stress occurs when there is an imbalance between the production of harmful molecules called free radicals and the body’s ability to neutralize them with antioxidants^50^. Prolonged oxidative stress is associated with various health issues, including non-communicable diseases (NCDs) such as cardiovascular diseases, diabetes, cancer, and neurodegenerative disorders^51^. Moreover, it has also been reported that vitamins, carotenoids, and other bioactive compounds are also present in different concentration in germinated grains other than polyphenol compounds and phenolic acids, which may function as additional antioxidants and significantly enhances the antioxidant activity of the grains during germination^52^. This, therefore, could explain the reason for the observed higher antioxidant activity in the germinated samples, although the samples recorded lower total phenolic content.

## Conclusion

Germination improved the biochemical composition of pigeon pea grains, which positively influenced the functional properties of flour, such as oil and water binding capacities, foaming capacity and bulk densities. Further, it enhanced the crude protein and total ash contents that contributed to the high nutritional value of the flour. The germinated pigeon pea flour possesses antioxidant potential and could be useful against free radical-induced disorders.

Based on the enhanced functional properties, germinated flour may be suitable for preparing innovative bakery products. Besides, the germinated flours could be used in cereal-based diets to address protein deficiency in developing countries. This will help promote the utilization of underutilized pigeon pea by incorporating them for the development of functional food products with high nutritional value and health-promoting bioactive compounds. The current investigation did not involve the creation of tangible food products to assess the applicability of germinated flour in such contexts. This aspect is recognised as a constraint in our study. Nevertheless, though this limitation might influence the breadth and understanding of our results, it also opens avenues for forthcoming research to confront these restrictions and expand upon our study's foundation.

## Methods

### Study area

The pigeon pea grains were purchased from the open market in the Tamale Metropolis in the Northern region of Ghana. The study of physico-functional and proximate properties was conducted at the Spanish Laboratory complex of the University for Development Studies, Nyankpala campus, Tamale, Ghana.

### Sample preparation

**Figure 5:**
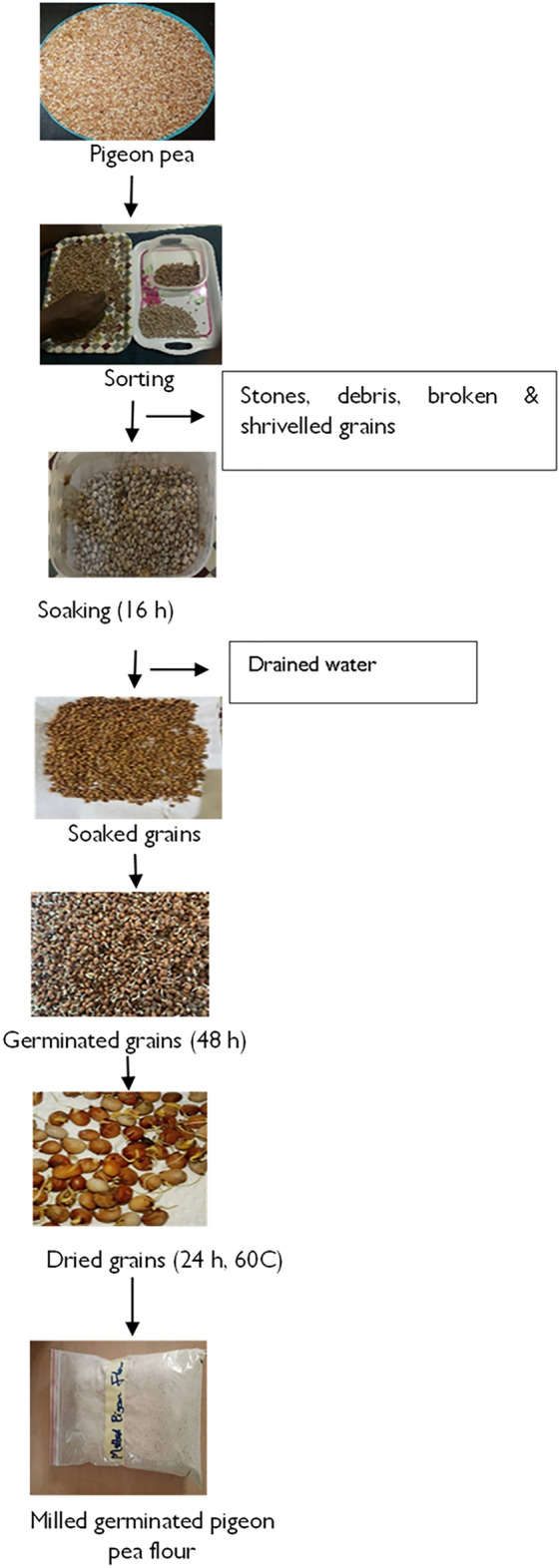
Preparation of germinated pigeon pea flour.

The grains, 5 kg, were first sorted to make sure all foreign materials and broken grains were removed. The germination of the pigeon pea was done following previous method^26^ as shown in Fig. 5.

### Physico-functional properties

#### pH

The pH of the pigeon pea flour samples was determined as described by the method of AOAC (2005)^53^ using the pH meter (JENWAY model: 3510).

#### Tapped bulk density (TBD)

The tapped bulk density was determined according to the method described by Abe-Inge et al.^54^. Briefly, 20 g of the flour samples were put into a 50-ml measuring cylinder. The cylinder was gently tapped on the benchtop 10 times from a height of 5 m from the ground. The bulk density was calculated as the weight per unit volume of the sample and computed as follows:

$${\text{TBD }}\;{\text{g}}/{\text{ml }} = \frac{{{\text{Weight }}\;{\text{of}}\;{\text{ Sample }}}}{{{\text{The }}\;{\text{volume }}\;{\text{of }}\;{\text{the }}\;{\text{sample }}\;{\text{after }}\;{\text{tapping}}}}$$

#### Foaming capacity

Foaming capacity was determined according to the method described elsewhere^55^. Approximately, 2 g of flour sample were weighed and added to 50 ml distilled water in a 100 ml measuring cylinder, The suspension was mixed and properly shaken to foam and the total volume after 30 s was recorded. The percentage increase in volume after 30 s is expressed as foaming capacity

$${\text{Foam}}\;{\text{ Capacity}}\% = \;\frac{{{\text{Volume}}\;{\text{ of}}\;{\text{ foam}}\;{\text{ after }}\;{\text{whipping}} - {\text{Volume }}\;{\text{of}}\;{\text{ foam}}\;{\text{ after }}\;{\text{whipping}}}}{{{\text{Volume }}\;{\text{of }}\;{\text{foam }}\;{\text{after }}\;{\text{whipping}}}} \times 100$$

#### Swelling index determination

The swelling capacity of the flour samples was determined by a modification of Onwuka^56^ method. Two (2) grammes of the sample were weighed into a measuring cylinder, and the volume was recorded. Ten (10) milliliters of distilled water were then added to each sample and the solution was allowed to stand for 30 min at room temperature (25 °C). The volume of each sample was retaken and recorded. The index of the swelling ability of the sample was calculated using the equation below

$${\text{Swelling capacity }} = \frac{Final volume of sample after swelling}{{\text{ Initial volume of sample }}}$$

#### Water and oil absorption capacity

Water- and oil absorption capacity of samples were carried out as reported elsewhere by Elkhalifa et al.^57^. Two grammes of each flour sample was weighed into a pre-weighed centrifuge tube and 20 ml of distilled water were added. For oil binding, 20 ml sunflower oil was added. Samples were vortexed and allowed to stand for 30 min at room temperature before being centrifuged at 4000 rpm for 25 min. Excess water or oil was decanted by inverting the tubes over absorbent paper and samples were allowed to drain. The weights of water and bound oil samples were determined by difference.

#### Proximate analysis

The methods described in the Official Methods of Analysis of the Association of Official Analytical Chemists (AOAC) International^58^ were used to determine the moisture (AOAC 925.10) with some slight modification by drying the samples at 105 °C overnight for approximately 12 h instead of 24 h, crude protein (AOAC 960.52), Ash (923.03) and crude fat (AOAC 922.06). Total carbohydrate was computed by difference.

#### Color determination

The color of the germinated pigeon pea flour was determined with a handheld Chromameter (Model: Konica Minolta CR-410) as earlier described^59^. Briefly, the Chroma meter was first calibrated with a white tile. Each flour sample was poured to fill a petri dish and then covered. The lens of the Chroma meter was placed on the petri dish at three different parts and the color was the taken. L* denotes darkness/lightness (0 = black, 100 = white), a* (− a = greenness and + a = redness), and b* (− b = blueness, + b = yellowness.

#### Total polyphenols content

In this study 100% methanol was used as solvent to extract the polyphenols in the pigeon pea flour samples. The total polyphenols content of the fermented flour samples was determined using the method developed in a previous study^60^. In brief, 1 ml of each sample extract was transferred into a 25 ml volumetric flask containing 2.5 ml of 3.54 g/l Iron (III) chloridehexahydrate solution. The volumetric flask containing the sample solution was then placed in a water bath and maintained at 80 ˚C for 20 min. After this, 2.5 ml of acetate buffer solution pH (4.6), 5.0 ml of 3.28 g/l 1,10-phenanthrolinehydrate (1,10-phen) and 2.5 ml of 3.72 g/l ethylene diaminetetraaceticaciddihydrate (EDTA) solutions were added, respectively. Finally, each flask was added. Finally, each flask was filled to the mark with distilled water, cooled and then the absorbance measurements were made at 511 nm.

The DPPH-free radical scavenging activity of the samples was determined according to previous work^61^. Approximately, 4 ml (0.004% w/v) of DPPH solution was mixed with 1.0 ml of extract (in methanol). The reaction mixture was vortex mixed thoroughly and incubated at room temperature in the dark for 30 min. Reduction in the absorbance of the mixture was measured at 517 nm using ascorbic acid as a control. Scavenging of DPPH radicals by the extract was calculated using the following formula:

$$\% {\text{ DPPH}}\;{\text{ free }}\;{\text{radical}}\;{\text{ scavenging}}\; \, = \;\frac{Absorbance \;of\; control - Absorbance \;of\; test}{{Absorbance \;of\; control}} \times 100$$

### Statistical analyses

Data on physico-functional, proximate and total phenolic content was analyzed using two-sample t-test in Minitab. The antioxidant activity of the samples was also analyzed using a one-way Analysis of Variance (ANOVA) to compare the means of the germinated flour, raw flours and the standard (ascorbic acid). The Fisher’s Least Significant Difference test was used to compare differences between means when the ANOVA result was significant (p < 0.05) (Supplementary file).

### Supplementary Information

Supplementary Information.

## Data Availability

The datasets generated during and/or analyzed during the current study are available from the corresponding author on reasonable request.
