# Supplementary material for: Physico-functional and nutritional characteristics of germinated pigeon pea (Cajanus cajan) flour as a functional food ingredient
Source: Sci Rep. 2023 Oct 3;13:16627. doi: 10.1038/s41598-023-43607-8 (PMC10547838; doi:10.1038/s41598-023-43607-8)

WORKSHEET 1

Two-Sample T-Test and CI: Moisture, Treatment

Method

μ_1: population mean of Moisture when Treatment = Malted
μ_2: population mean of Moisture when Treatment = Raw
Difference: $\mu_1 - \mu_2$

Equal variances are not assumed for this analysis.

Descriptive Statistics: Moisture

Treatment	N	Mean	StDev	SE Mean
Malted	3	10.2313	0.0845	0.049
Raw	3	12.219	0.264	0.15

Estimation for Difference

Difference	95% CI for Difference
-1.988	(-2.676, -1.300)

Test

Null hypothesis $H_0: \mu_1 - \mu_2 = 0$
Alternative hypothesis $H_1: \mu_1 - \mu_2 \neq 0$

T-Value	DF	P-Value
-12.43	2	0.006

WORKSHEET 1

Two-Sample T-Test and CI: Crude Fat (DMB), Treatment

Method

μ_1: population mean of Crude Fat (DMB) when Treatment = Malted
μ_2: population mean of Crude Fat (DMB) when Treatment = Raw
Difference: $\mu_1 - \mu_2$

Equal variances are not assumed for this analysis.

Descriptive Statistics: Crude Fat (DMB)

Treatment	N	Mean	StDev	SE Mean
Malted	3	1.6177	0.0640	0.037
Raw	3	1.8659	0.0338	0.020

Estimation for Difference

Difference	95% CI for Difference
-0.2483	(-0.3812, -0.1153)

Test

Null hypothesis $H_0: \mu_1 - \mu_2 = 0$
Alternative hypothesis $H_1: \mu_1 - \mu_2 \neq 0$

T-Value	DF	P-Value
-5.94	3	0.010

WORKSHEET 1

Two-Sample T-Test and CI: Total ash (DMB), Treatment

Method

μ_1: population mean of Total ash (DMB) when Treatment = Malted
μ_2: population mean of Total ash (DMB) when Treatment = Raw
Difference: $\mu_1 - \mu_2$

Equal variances are not assumed for this analysis.

Descriptive Statistics: Total ash (DMB)

Treatment	N	Mean	StDev	SE Mean
Malted	3	4.2140	0.0257	0.015
Raw	3	3.6111	0.0542	0.031

Estimation for Difference

Difference	95% CI for Difference
0.6029	(0.4539, 0.7519)

Test

Null hypothesis	$H_0: \mu_1 - \mu_2 = 0$
Alternative hypothesis	$H_1: \mu_1 - \mu_2 \neq 0$

T-Value	DF	P-Value
17.41	2	0.003

WORKSHEET 1

Two-Sample T-Test and CI: Crude prot (DMB), Treatment

Method

μ_1: population mean of Crude prot (DMB) when Treatment = Malted
μ_2: population mean of Crude prot (DMB) when Treatment = Raw
Difference: $\mu_1 - \mu_2$

Equal variances are not assumed for this analysis.

Descriptive Statistics: Crude prot (DMB)

Treatment	N	Mean	StDev	SE Mean
Malted	3	11.580	0.830	0.48
Raw	3	9.378	0.829	0.48

Estimation for Difference

Difference	95% CI for Difference
2.201	(0.047, 4.356)

Test

Null hypothesis	$H_0: \mu_1 - \mu_2 = 0$
Alternative hypothesis	$H_1: \mu_1 - \mu_2 \neq 0$

T-Value	DF	P-Value
3.25	3	0.047

WORKSHEET 1

Two-Sample T-Test and CI: Total CHO, Treatment

Method

μ_1: population mean of Total CHO when Treatment = Malted
μ_2: population mean of Total CHO when Treatment = Raw
Difference: $\mu_1 - \mu_2$

Equal variances are not assumed for this analysis.

Descriptive Statistics: Total CHO

Treatment	N	Mean	StDev	SE Mean
Malted	3	72.357	0.874	0.50
Raw	3	72.93	1.05	0.61

Estimation for Difference

Difference	95% CI for Difference

-0.568	(-3.076, 1.939)

Test

Null hypothesis $H_0: \mu_1 - \mu_2 = 0$
Alternative hypothesis $H_1: \mu_1 - \mu_2 \neq 0$

T-Value	DF	P-Value
-0.72	3	0.523

WORKSHEET 1

Two-Sample T-Test and CI: pH, Treatment

Method

μ_1: population mean of pH when Treatment = Malted
μ_2: population mean of pH when Treatment = Raw
Difference: $\mu_1 - \mu_2$

Equal variances are not assumed for this analysis.

Descriptive Statistics: pH

Treatment	N	Mean	StDev	SE Mean
Malted	3	4.9167	0.0231	0.013
Raw	3	3.773	0.225	0.13

Estimation for Difference

Difference	95% CI for Difference
1.143	(0.582, 1.705)

Test

Null hypothesis $H_0: \mu_1 - \mu_2 = 0$
Alternative hypothesis $H_1: \mu_1 - \mu_2 \neq 0$

T-Value	DF	P-Value
8.76	2	0.013

WORKSHEET 1

Two-Sample T-Test and CI: Bulk Density, Treatment

Method

μ_1: population mean of Bulk Density when Treatment = Malted
μ_2: population mean of Bulk Density when Treatment = Raw
Difference: $\mu_1 - \mu_2$

Equal variances are not assumed for this analysis.

Descriptive Statistics: Bulk Density

Treatment	N	Mean	StDev	SE Mean
Malted	3	0.5484	0.0135	0.0078
Raw	3	0.62600	0.00556	0.0032

Estimation for Difference

Difference	95% CI for Difference
-0.07759	(-0.11375, -0.04142)

Test

Null hypothesis $H_0: \mu_1 - \mu_2 = 0$
Alternative hypothesis $H_1: \mu_1 - \mu_2 \neq 0$

T-Value	DF	P-Value
-9.23	2	0.012

WORKSHEET 1

Two-Sample T-Test and CI: Tapped Density, Treatment

Method

μ_1: population mean of Tapped Density when Treatment = Malted
μ_2: population mean of Tapped Density when Treatment = Raw
Difference: $\mu_1 - \mu_2$

Equal variances are not assumed for this analysis.

Descriptive Statistics: Tapped Density

Treatment	N	Mean	StDev	SE Mean
Malted	3	0.83917	0.00850	0.0049
Raw	3	0.77766	0.00590	0.0034

Estimation for Difference

Difference	95% CI for Difference
0.06152	(0.04250, 0.08053)

Test

Null hypothesis $H_0: \mu_1 - \mu_2 = 0$
Alternative hypothesis $H_1: \mu_1 - \mu_2 \neq 0$

T-Value	DF	P-Value
10.30	3	0.002

WORKSHEET 1

Two-Sample T-Test and CI: %Swelling Index, Treatment

Method

μ_1: population mean of %Swelling Index when Treatment = Malted
μ_2: population mean of %Swelling Index when Treatment = Raw
Difference: $\mu_1 - \mu_2$

Equal variances are not assumed for this analysis.

Descriptive Statistics: %Swelling Index

Treatment	N	Mean	StDev	SE Mean
Malted	3	258.8	15.2	8.8
Raw	3	272.90	8.83	5.1

Estimation for Difference

Difference	95% CI for Difference
-14.1	(-46.4, 18.2)

Test

Null hypothesis $H_0: \mu_1 - \mu_2 = 0$
Alternative hypothesis $H_1: \mu_1 - \mu_2 \neq 0$

T-Value	DF	P-Value
-1.39	3	0.259

WORKSHEET 1

Two-Sample T-Test and CI: %OAC, Treatment

Method

μ_1: population mean of %OAC when Treatment = Malted
μ_2: population mean of %OAC when Treatment = Raw
Difference: $\mu_1 - \mu_2$

Equal variances are not assumed for this analysis.

Descriptive Statistics: %OAC

Treatment	N	Mean	StDev	SE Mean
Malted	3	237.55	5.92	3.4
Raw	3	219.90	3.78	2.2

Estimation for Difference

Difference	95% CI for Difference
17.64	(4.73, 30.55)

Test

Null hypothesis	$H_0: \mu_1 - \mu_2 = 0$
Alternative hypothesis	$H_1: \mu_1 - \mu_2 \neq 0$

T-Value	DF	P-Value
4.35	3	0.022

WORKSHEET 1

Two-Sample T-Test and CI: %FC, Treatment

Method

μ_1: population mean of %FC when Treatment = Malted
μ_2: population mean of %FC when Treatment = Raw
Difference: $\mu_1 - \mu_2$

Equal variances are not assumed for this analysis.

Descriptive Statistics: %FC

Treatment	N	Mean	StDev	SE Mean
Malted	3	55.333	0.577	0.33
Raw	3	52.00	1.00	0.58

Estimation for Difference

Difference	95% CI for Difference
3.333	(1.212, 5.455)

Test

Null hypothesis	$H_0: \mu_1 - \mu_2 = 0$
Alternative hypothesis	$H_1: \mu_1 - \mu_2 \neq 0$

T-Value	DF	P-Value
5.00	3	0.015

WORKSHEET 1

Two-Sample T-Test and CI: %WAC, Treatment

Method

μ_1: population mean of %WAC when Treatment = Malted
μ_2: population mean of %WAC when Treatment = Raw
Difference: $\mu_1 - \mu_2$

Equal variances are not assumed for this analysis.

Descriptive Statistics: %WAC

Treatment	N	Mean	StDev	SE Mean
Malted	3	277.0	13.5	7.8
Raw	3	265.45	3.82	2.2

Estimation for Difference

Difference	95% CI for Difference
11.52	(-23.39, 46.43)

Test

Null hypothesis	$H_0: \mu_1 - \mu_2 = 0$
Alternative hypothesis	$H_1: \mu_1 - \mu_2 \neq 0$

T-Value	DF	P-Value
1.42	2	0.292

WORKSHEET 1

Two-Sample T-Test and CI: L*, Sample ID

Method

μ_1: population mean of L* when Sample ID = Malted Pigeon Pea
μ_2: population mean of L* when Sample ID = Raw Pigeon Pea
Difference: $\mu_1 - \mu_2$

Equal variances are not assumed for this analysis.

Descriptive Statistics: L*

Sample ID	N	Mean	StDev	SE Mean
Malted Pigeon Pea	3	70.77	3.48	2.0
Raw Pigeon Pea	3	48.777	0.741	0.43

Estimation for Difference

Difference	95% CI for Difference
21.99	(13.15, 30.84)

Test

Null hypothesis	$H_0: \mu_1 - \mu_2 = 0$
Alternative hypothesis	$H_1: \mu_1 - \mu_2 \neq 0$

T-Value	DF	P-Value
10.70	2	0.009

WORKSHEET 1

Two-Sample T-Test and CI: a, Sample ID

Method

μ_1: population mean of a when Sample ID = Malted Pigeon Pea
μ_2: population mean of a when Sample ID = Raw Pigeon Pea
Difference: $\mu_1 - \mu_2$

Equal variances are not assumed for this analysis.

Descriptive Statistics: a

Sample ID	N	Mean	StDev	SE Mean
Malted Pigeon Pea	3	2.693	0.429	0.25
Raw Pigeon Pea	3	5.677	0.635	0.37

Estimation for Difference

Difference	95% CI for Difference
-2.983	(-4.392, -1.575)

Test

Null hypothesis	$H_0: \mu_1 - \mu_2 = 0$
Alternative hypothesis	$H_1: \mu_1 - \mu_2 \neq 0$

T-Value	DF	P-Value
-6.74	3	0.007

WORKSHEET 1

Two-Sample T-Test and CI: b*, Sample ID

Method

μ_1: population mean of b* when Sample ID = Malted Pigeon Pea
μ_2: population mean of b* when Sample ID = Raw Pigeon Pea
Difference: $\mu_1 - \mu_2$

Equal variances are not assumed for this analysis.

Descriptive Statistics: b*

Sample ID	N	Mean	StDev	SE Mean
Malted Pigeon Pea	3	21.820	0.639	0.37
Raw Pigeon Pea	3	17.263	0.296	0.17

Estimation for Difference

Difference	95% CI for Difference
4.557	(2.807, 6.306)

Test

Null hypothesis $H_0: \mu_1 - \mu_2 = 0$
Alternative hypothesis $H_1: \mu_1 - \mu_2 \neq 0$

T-Value	DF	P-Value
11.20	2	0.008

WORKSHEET 1

Two-Sample T-Test and CI: c*, Sample ID

Method

μ_1: population mean of c* when Sample ID = Malted Pigeon Pea
μ_2: population mean of c* when Sample ID = Raw Pigeon Pea
Difference: $\mu_1 - \mu_2$

Equal variances are not assumed for this analysis.

Descriptive Statistics: c*

Sample ID	N	Mean	StDev	SE Mean
Malted Pigeon Pea	3	21.993	0.628	0.36
Raw Pigeon Pea	3	18.177	0.280	0.16

Estimation for Difference

Difference	95% CI for Difference
3.817	(2.109, 5.524)

Test

Null hypothesis $H_0: \mu_1 - \mu_2 = 0$
Alternative hypothesis $H_1: \mu_1 - \mu_2 \neq 0$

T-Value	DF	P-Value
9.62	2	0.011

WORKSHEET 1

Two-Sample T-Test and CI: h, Sample ID

Method

μ_1: population mean of h when Sample ID = Malted Pigeon Pea
μ_2: population mean of h when Sample ID = Raw Pigeon Pea
Difference: $\mu_1 - \mu_2$

Equal variances are not assumed for this analysis.

Descriptive Statistics: h

Sample ID	N	Mean	StDev	SE Mean
Malted Pigeon Pea	3	82.96	1.15	0.66
Raw Pigeon Pea	3	71.80	2.04	1.2

Estimation for Difference

Difference	95% CI for Difference
11.16	(6.86, 15.45)

Test

Null hypothesis	H_0: μ_1 - μ_2 = 0
Alternative hypothesis	H_1: μ_1 - μ_2 ≠ 0

T-Value	DF	P-Value
8.27	3	0.004

WORKSHEET 1

Two-Sample T-Test and CI: TPC (mg GAE/ml), Treatment

Method

μ_1: population mean of TPC (mg GAE/ml) when Treatment = Germinated
μ_2: population mean of TPC (mg GAE/ml) when Treatment = Raw
Difference: μ_1 - μ_2

Equal variances are not assumed for this analysis.

Descriptive Statistics: TPC (mg GAE/ml)

Treatment	N	Mean	StDev	SE Mean
Germinated	3	0.0709	0.0152	0.0088
Raw	3	0.08913	0.00580	0.0033

Estimation for Difference

Difference	95% CI for Difference
-0.01820	(-0.05863, 0.02223)

Test

Null hypothesis	H_0: μ_1 - μ_2 = 0
Alternative hypothesis	H_1: μ_1 - μ_2 ≠ 0

T-Value	DF	P-Value
-1.94	2	0.192

WORKSHEET 2

One-way ANOVA: %RSA versus Treatment

Method

Null hypothesis	All means are equal
Alternative hypothesis	Not all means are equal
Significance level	α = 0.05

Equal variances were assumed for the analysis.

Factor Information

Factor	Levels	Values
Treatment	3	Germinated, Raw, Standard

Analysis of Variance

Source	DF	Adj SS	Adj MS	F-Value	P-Value
Treatment	2	2858.23	1429.11	214.44	0.000
Error	6	39.99	6.66		
Total	8	2898.21			

Model Summary

S	R-sq	R-sq(adj)	R-sq(pred)
2.58156	98.62%	98.16%	96.90%

Means

Treatment	N	Mean	StDev	95% CI
Germinated	3	94.511	0.578	(90.864, 98.158)
Raw	3	58.27	4.43	(54.62, 61.92)
Standard	3	97.4640	0.1493	(93.8170, 101.1110)

Pooled StDev = 2.58156

Fisher Pairwise Comparisons

Grouping Information Using the Fisher LSD Method and 95% Confidence

Treatment	N	Mean	Grouping	
Standard	3	97.4640	A	
Germinated	3	94.511	A	
Raw	3	58.27		B

Means that do not share a letter are significantly different.

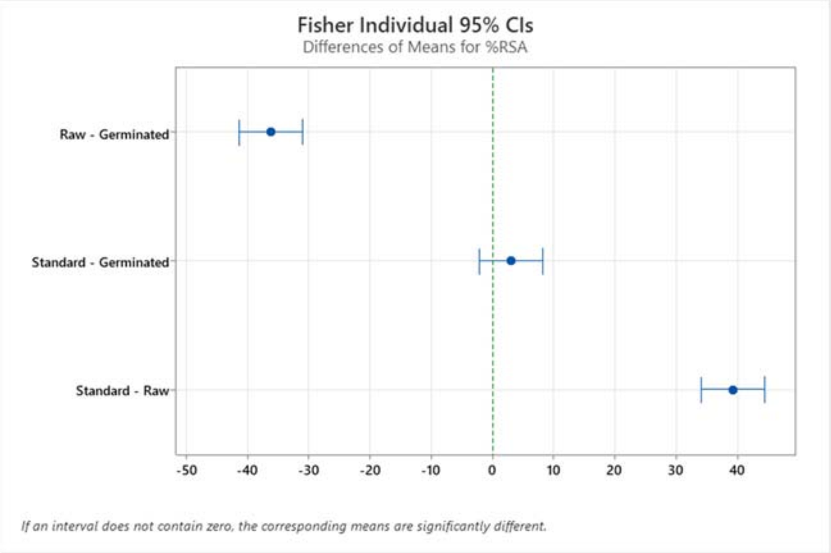

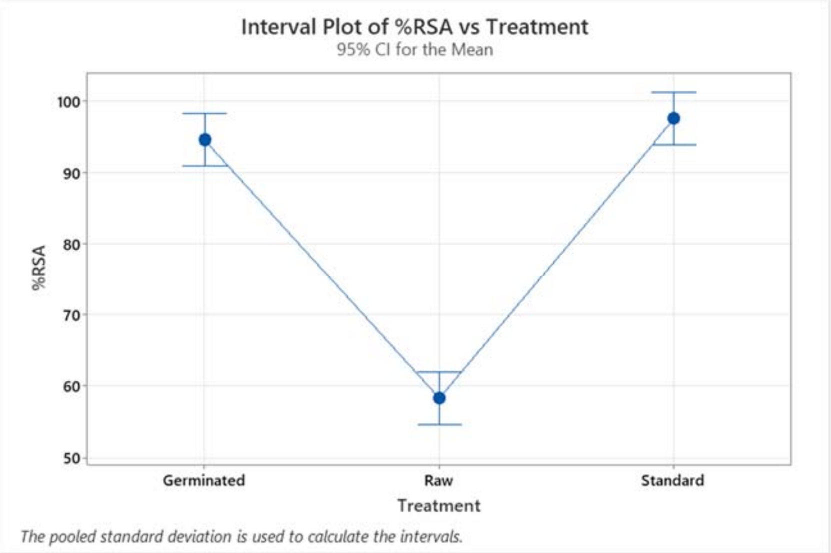

Supplement: Supplementary file 1 — Supplementary Information. [file 41598_2023_43607_MOESM1_ESM.pdf]
